# Just how Lamarckian is CRISPR-Cas immunity: the continuum of evolvability mechanisms

**DOI:** 10.1186/s13062-016-0111-z

**Published:** 2016-02-24

**Authors:** Eugene V. Koonin, Yuri I. Wolf

**Affiliations:** National Center for Biotechnology Information, National Library of Medicine, National Institute of Health, Bethesda, MD 20894 USA

**Keywords:** CRISPR-Cas, Self-nonself discrimination, Lamarckian evolution, Darwinian evolution, DNA repair

## Abstract

The CRISPR-Cas system of prokaryotic adaptive immunity displays features of a mechanism for directional, Lamarckian evolution. Indeed, this system modifies a specific locus in a bacterial or archaeal genome by inserting a piece of foreign DNA into a CRISPR array which results in acquired, heritable resistance to the cognate selfish element. A key element of the Lamarckian scheme is the specificity and directionality of the mutational process whereby an environmental cue causes only mutations that provide specific adaptations to the original challenge. In the case of adaptive immunity, the specificity of mutations is equivalent to self-nonself discrimination. Recent studies on the CRISPR mechanism have shown that the levels of discrimination can substantially differ such that in some CRISPR-Cas variants incorporation of DNA is random whereas discrimination occurs by selection of cells that carry cognate inserts. In other systems, a higher level of specificity appears to be achieved via specialized mechanisms. These findings emphasize the continuity between random and directed mutations and the critical importance of evolved mechanisms that govern the mutational process.

**Reviewers:** This article has been reviewed by Yitzhak Pilpel, Martijn Huynen, and Bojan Zagrovic.

## Background

Inheritance of Acquired adaptive Characters (IAC) has been a subject of thorny, circuitous debates among biologists over the last two centuries [1]. Jean-Bapteste Lamarck, the first scholar to propose a coherent account of biological evolution, considered IAC to be the primary if not the only route of evolutionary change, even if he did not claim the idea of IAC as his own but rather as almost common knowledge of the time [2, 3]. Charles Darwin emphasized random heritable changes [4] but in his later thinking, reflected in the last editions of the *Origin of Species*, was inclined to attribute a substantial role to IAC as well, apparently under the weight of doubts regarding the sufficiency of random changes as the only source of evolutionarily relevant variation [5]. Thousands of subsequent experiments, most notably the fluctuation test of Luria and Delbruck, have revealed the dominance of random mutations [6, 7]. For several decades in the 20^th^ century, IAC has fallen out of fashion in mainstream biology, and worse, has been central to several pseudo-scientific fads the foremost of which was the infamous Lysenkoism [8, 9]. However, over the last few decades, an increasing number of findings on apparent directional, adaptive mutations as well as heritable epigenetic changes apparently directly caused by environmental factors have suggested partial rehabilitation of IAC [10–12].

Among the genetic phenomena that might involve IAC, the prokaryotic adaptive immunity mediated by CRISPR-Cas (Clustered Regularly Interspaced Short Palindromic Repeats- CRISPR-ASsociated genes) systems is arguably the most compelling case [10–12]. The CRISPR-Cas immune response involves insertion of pieces of foreign DNA, such as a viral or plasmid genome, specifically into the CRISPR array (these inserts are denoted spacers because they are positioned between repeats in the CRISPR array; the sequences in the foreign DNA that give rise to spacers are accordingly denoted protospacers, and this first stage of the CRISPR immune response is known as adaptation), followed by utilization of the processed CRISPR transcript (crRNA) as guides for inactivation of the cognate target [13–19]. The net result is the acquired, heritable, highly specific and efficient protection against the cognate (parasitic) element. Characteristic of immune mechanisms coevolving with parasites, the CRISPR-Cas systems show extreme diversity, with 6 distinct types and 19 subtypes identified on the basis of protein domain compositions and genomic loci architectures [20, 21].

**Fig. 1:**
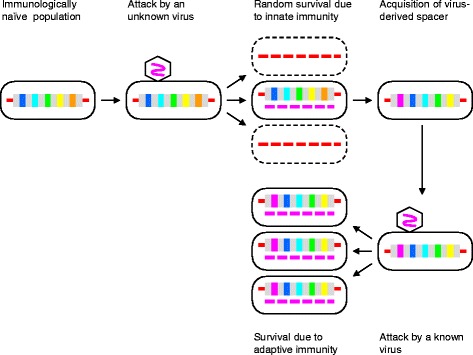
The Lamarckian scenario for the CRISPR immune response: efficient self-nonself discrimination

Phenomenologically, the CRISPR-mediated immunity has all the ingredients of IAC, or Lamarckian evolution: the genome of a bacterium or archaeon is modified in a highly specific manner, in response to a specific environmental challenge (such as virus infection), resulting in a highly specific and efficient adaptation to that particular challenge (Fig. 1) [11]. The realization of the apparent Lamarckian character of the CRISPR-mediated immunity stimulated examination of many other phenomena that involve seemingly non-random genomic changes from the perspective of IAC. As a result, several processes, such as stress-induced mutagenesis and certain types of horizontal gene transfer have been classified as “quasi-Lamarckian” [10, 11].

Whether or not a particular process qualifies as a bona fide case of IAC or Lamarckian evolution, hinges on the specificity of the mutations involved. Traditionally, the concept of the Lamarckian mechanism of evolution is predicated on a high specificity of mutations, i.e., only the mutations that are adaptive with respect to the respective causative factor are supposed to occur. In the case of an adaptive immune system, such as CRISPR-Cas, this requirement boils down to the fidelity of self-nonself discrimination. Several recent observations indicate that CRISPR-Cas systems differ from each other in that respect so that the specificity toward foreign target DNAs is at least in part determined by selection. Here we discuss the implications of these findings for understanding the CRISPR-Cas mechanisms and more generally for the IAC problem.

## Self-nonself discrimination in CRISPR-Cas systems

For CRISPR-Cas systems, self-nonself discrimination is relevant at two levels. First, discrimination obviously is essential at the interference stage: the CRISPR machinery must not target the spacer itself within the repeat array. Obviously, such targeting would typically cause cell death. Most of the CRISPR-Cas systems avoid this outcome through the requirement for the so-called Protospacer Adjacent Motif (PAM), a short sequence next to the protospacer that is recognized by the adaptation complex and is essential for spacers acquisition [22–26]. Although the PAM is a short, partially redundant sequence signature, it is strictly avoided in the CRISPR, thus preventing self-destruction [27]. Type III CRISPR-Cas systems appear not to require a PAM and instead apparently avoid self-targeting due to the requirement of non-complementarity between the crRNA and the target DNA in the sequence adjacent to the spacer which could be an additional safeguard against self-destruction in all CRISPR-cas systems [28].

Second, discrimination involves distinguishing between foreign and host DNA at the adaptation stage. Outside the specific context of the CRISPR array, the PAM cannot provide for efficient self-nonself discrimination because at any given information content of the motif, the host genome, being much larger than the genome of the targeted selfish element, is overwhelmingly likely to contain many more copies of the PAM. Increasing the size and specificity of the PAM and selecting the host for depletion of the PAM occurrence would have a deleterious effect because in this case, many genomes of selfish elements, especially small ones, would contain no or too few copies of the PAM to allow efficient adaptation and protection.

Despite the lack of an obvious discrimination mechanism, the initial analyses of CRISPR spacers have identified a small fraction of sequences that were (nearly) identical to fragments of phage or plasmid genomes whereas the rest of the spacers showed no significant similarity to any available sequences [29–31]. Follow-up analyses have identified some bacterial genomes that contained a higher fraction of phage-matching spacers [32]. The inference from these observations was that the immune system discriminates between self (the respective bacterial or archaeal DNA) and nonself (foreign DNA) with high fidelity, whereas the “orphan” spacers either represent the still unchartered diversity of mobile elements or fail to match such elements due to escape mutations in the latter. Subsequently, a few spacers have been discovered that matched the host genome, leading to the natural idea that autoimmunity could emerge as a consequence of errors in the discrimination [33, 34]. However, these findings have been made on spacers that were fixed in the microbial population or at least have spread through thousands of cell divisions. Recent unbiased analyses of the process of spacer acquisition yield a more complex picture. In an assay for spacer acquisition by the type I-E CRISPR-Cas system of *Escherichia coli* where the experimental setup prevented cell killing by self-targeting spacers, a substantial excess of spacers from plasmid DNA over those from chromosomal DNA was observed [35].

**Fig. 2:**
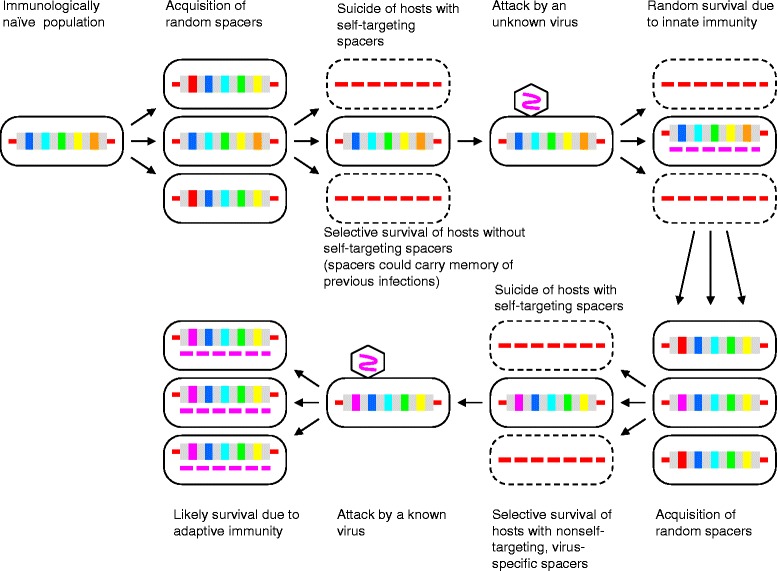
The Darwinian scenario for the CRISPR immune response: random spacer acquisition with subsequent selection

In contrast, experiments with the type II-A CRISPR-Cas system from *Streptococcus thermophilus* provide evidence of apparently random spacer acquisition [36]. When the nuclease activity of the endonuclease Cas9 is knocked out and the suicidal effect of autoimmunity is accordingly prevented, the overwhelming majority of the inserted spacers were from the host genome. The implication of this experiment is as startling as it is obvious: apparently, in this case, the CRISPR-Cas system is extremely wasteful, with the majority of cells committing suicide, so that upon an attack by a selfish element, the few that incorporate spacers homologous to the invader genome could survive (Fig. 2).

A breakthrough recent study on spacer acquisition by the *E. coli* type I-E CRISPR-cas system has revealed a 100-1000 excess of foreign over host DNA among the inserted spacers and reported the first substantial clues into the discrimination mechanisms [37]. Specifically, it has been shown that spacer acquisition requires active replication of the protospacer-containing DNA, with spacers being acquired primarily at stalled replication forks. Accordingly, small, fast replicating plasmid genomes are much more efficient as a sources of spacers than the host DNA. These findings are compatible with earlier observations in the archaeon *Sulfolobus* islandicus which indicate that acquisition of spacers from an infecting virus genome required its active replication [38]. Further experiments have shown that at least in *E. coli*, the regions of active spacer acquisition lie between a stalled replication fork and a *Chi* site [39], and acquisition is about 10-fold reduced in RecB,C,D mutants. Thus, it appears most likely that in this system, spacers are primarily derived from products of RecBCD-catalyzed DNA degradation that are produced during the repair of double-stranded breaks associated with stalled replication forks. These experiments seem to reveal at least one mechanism of self-nonself discrimination by the CRISPR-Cas machinery that is not based on any intrinsic differences between foreign and host DNA but rather on the much greater density of replication forks, and accordingly, double-stranded breaks in the former [40].

Another remarkable mechanism of self-nonself discrimination by the type I-E CRISPR-Cas system involves the phenomenon dubbed priming whereby the acquisition of spacers from DNA containing at least one (partial) match to pre-existing spacers in the given host is strongly stimulated compared to the acquisition from DNA devoid of such sequences [41–44]. Unlike unprimed acquisition, which depends only on Cas1 and Cas2 proteins, priming requires the involvement of the entire set of Cas protein. Thus, it appears that, after recognizing the cognate protospacer, the Cas machinery (the Cascade complex) efficiently generates new spacers, apparently without dissociating from the target DNA and without a strict the requirement for the PAM. Regardless of the details of the mechanism that remain to be elucidated, the net outcome is a strong enhancement of self-nonself discrimination.

Apart from the replication-dependent discrimination and priming, at least some CRISPR-Cas systems are normally repressed and are induced only upon infection, thus further curtailing the deleterious effect of autoimmunity [45].

## Inheritance of acquired characters, biased mutation, selection and evolvability mechanisms

The recent findings outlined above suggest that different types of CRISRP-Cas systems substantially differ with respect to the efficiency (and possibly even existence) of their mechanisms for self-nonself discrimination. Given the extreme diversity of *cas* gene composition among the different types and subtypes of CRISPR-Cas [20], these results imply that observations on a particular CRISPR-Cas variant might not be readily transferable to other variants. Thus, much more experimentation on diverse archaeal and bacterial models is required to achieve full understanding of the immune specificity and mechanisms. Nevertheless, even the already available results have potentially important implications for understanding the status of IAC in genome evolution.

The apparent lack of self-nonself discrimination in type II-A CRISPR-Cas pushes this systems into the domain of “quasi-Lamarckian” phenomena [10, 11] or perhaps outside the Lamarckian domain altogether. Indeed, this system seems to rely on a “semi-random” insertional mutagenesis where the insertion site is strictly determined (restricted to the CRISPR array) but the inserted sequences (spacers) are random. Moreover, these mutations are deleterious due to autoimmunity but the power of selection for resistance to virus infection that is provided by occasional beneficial mutations (insertions of viral DNA) is sufficient to maintain the CRISPR-Cas system during evolution (Fig. 2). The key ingredient of the Lamarckian process (IAC) is missing from this scenario, namely the direct induction of specific, adaptive mutations by the environmental challenge. Although this CRISPR-Cas systems directs the genomic changes (mutations) to the CRISPR array, these mutations are non-adaptive. Thus, the process seems to better fit the classical Darwinian scheme whereby the mutational process is largely random (and hence wasteful) whereas specificity and adaptation are achieved via selection. In a stark contrast, the type I-E CRISPR-Cas system seems to operate via a bona fide Lamarckian mechanism where the mutational process is dominated by directional, adaptive mutations which is achieved via the coupling of spacer acquisition with replication accompanied by the DSB formation and the priming mechanism.

The key message from these experiments could be the very existence of major differences in the mechanisms of different CRISPR-Cas systems such that some seem to be “Darwinian” whereas others appear “Lamarckian”. Subsequent experiments with other CRISPR-Cas varieties will determine how typical are the Lamarckian and Darwinian modalities, and if the latter could be caused by specific experimental conditions that somehow prevent discrimination. However, regardless of the outcome of these experiments, the demonstration that self-nonself discrimination is not inherent to CRISPR-Cas appears to be important in itself. The involvement of selection in the CRISPR-Cas response is not limited to self-nonself discrimination at the adaptation stage. It has been shown that the type II-A CRISPR-Cas systems of *S. thermophilus* initially incorporates numerous, diverse spacers of which only a few are subsequently selected and inherited through cell generations [46]. The criteria of selection and whether this is a common feature of different CRISPR-Cas systems remain unclear. Nevertheless, such observations indicate that at least some forms of the CRISPR-Cas response heavily depend on selection.

Despite many remaining uncertainties, taken together, the above results shed light on the different modes of the evolutionary process by showing what a fine line exists between the Darwinian and Lamarckian modalities and suggesting this line can be crossed on multiple occasions. The key feature of the Lamarckian mode is the non-randomness of mutations that is achieved via evolved mechanisms that are highly specific, elaborate and subject to regulation. Such mechanisms function in all apparent Lamarckian and quasi-Lamarckian evolutionary processes including CRISPR-Cas, the Piwi-RNA-based defense against transposons in animals, stress-induced mutagenesis and others [10, 11]. Furthermore, sophisticated mechanisms ensuring the specificity of heritable changes exist not only in genetic but also in epigenetic IAC. These mechanisms widely differ in their levels of specificity, from randomness to extremely high selectivity. Hence the continuum of evolutionary modes spanning the entire range from purely Darwinian and Wrightian (i.e., based on genetic drift) to bona fide Lamarckian. At a high level of abstraction, the multiplicity of mechanisms of partially specific evolvability fits the concept of read-write genome recently developed by Shapiro [47–49].

**Fig. 3:**
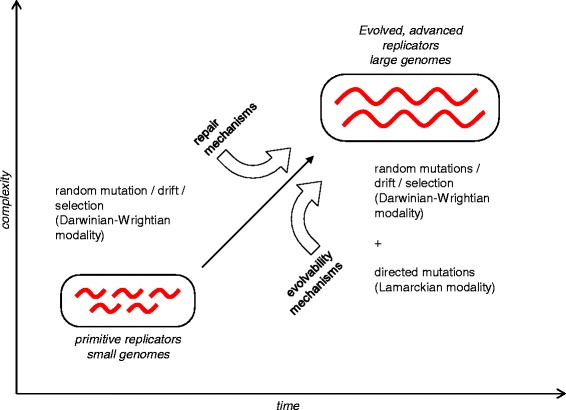
Evolution of mechanisms for repair and evolvability

Darwinian evolution that is based on negative and positive selection acting on random mutations as well as genetic drift (Wrightian evolution) are intrinsic features of replicator systems, hence in operation since the origin of the first replicators (that is, effectively, the origin of life) [10]. Evolution of life forms of increasing complexity was enabled by increasing replication fidelity through the evolution of repair mechanisms [50, 51]. The evolvability mechanisms resulting in (quasi)Lamarckian evolution seem to have evolved jointly with and in part as a by-product of the evolution of repair (Fig. 3). The two classes of mechanisms are tightly intertwined, functionally and evolutionarily. The apparent reliance of self-nonself discrimination in the CRISPR-Cas response on repair processes is discussed above [37]. Indeed, an early analysis of the Cas protein sequences and predicted functions has led to the hypothesis that these proteins together comprised a novel repair system, and not without a good reason because the repertoires of proteins in repair and CRISPR immunity (primarily, various nucleases and helicases) clearly overlap [52]. Moreover, it has been shown that knockout of the *E. coli cas1* gene leads to phenotypes deficient in various forms of repair [53]. Strong connections to repair also exist for other evolvability mechanisms leading to (quasi)Lamarckian phenomena, such as stress-induced mutagenesis and HGT.

The description of some forms of HGT and the CRISPR-Cas response as (quasi)Lamarckian phenomena has been criticized, first, because such phenomena only become apparent when the organismal level of selection is considered [54] and second, because historically, Lamarckian evolution implies a teleological aspect in evolution [55]. Both criticisms touch upon important aspects of the evolutionary process and, in our view, are answered by the present analysis. Indeed, the (quasi)Lamarckian phenomena are based on evolved mechanisms that could only emerge in relatively complex life forms such as first cells. Such mechanisms have nothing to do with teleology but evolved under the pressure to ensure efficient evolvability by biasing the mutational process and restricting mutations to specific parts of genomes.

## Conclusions

Several recent findings indicate that CRISPR-Cas systems of adaptive immunity differ with respect to the level of self-nonself discrimination at the adaptation stage. Some of these systems seem to acquire spacers randomly, resulting in extensive cell death, with subsequent selection of surviving cells that are resistant to infection thanks to the incorporation of spacers from parasite DNA. Other CRISPR-Cas variants possess mechanisms of efficient self-nonself discrimination during adaptation such that the incorporated spacers come almost entirely from foreign DNA. These systems seem to qualify as machines for Lamarckian evolution. Subsequent experiments will determine how common is each of these scenarios among the diverse CRISPR-cas systems in bacteria and archaea. However, even at this stage, it is becoming clear that the Darwinian and Lamarckian modes of evolution form a continuum of evolutionary regimes defined by mechanisms of evolvability that bias the mutational process to different degrees of specificity. In the course of evolution, Darwinian selection and genetic drift appeared first as intrinsic properties of replicators whereas the (quasi)Lamarckian mechanisms evolved jointly with mechanisms of DNA repair.

## Reviewers’ comments

### Reviewer 1: Yitzhak Pilpel, Weizmann Institute of Science

Reading this piece by Koonin and Wolf was very intellectually stimulating. I have enjoyed and learned a lot from it. I’d publish it as is, and look to see how it further affects the community. It did stimulate me to think further deeper into the problem. It was already appreciated in recent years that CRISPR-based immunity represents a form of Lamarckian evolution in bacteria, along with additional Lamarckian or quasi-Lamarckian means of evolution such as HGT, prions and more. Yet whether such mechanisms are truly Lamarckian, or in fact actually Darwinian, is now addressed through an interesting sieve. Concentrating predominantly on CRISPR, a distinction is made here between types of CRISPR systems that incorporate random DNA segments to serve as spacers, thus not distinguishing between self and nonself DNA, from other varieties of CRISPR, that are biased towards acquisition of nonself DNA, thus reducing the risk of auto-immunity. It is proposed here that only discriminatory CRISPR systems, of the later type, constitute truly Lamarckian, the others rely of Darwinian selection to eliminate auto-immunogenic events. The argument is well taken, and the distinction between the two evolutionary mechanisms is illuminating. The authors nicely build their case based on analyses on recent progress and breakthroughs in understanding how biased or random acquisition of spacers is obtained, thus providing a solid molecular mechanistic basis for their principled distinctions between the two modes of inheritance and evolution.

Towards the end of the piece the authors broaden the scope to discuss also non-CRISPR means of Lamarckian, or quasi-Lamarckian evolution. They suggest that in other cases too, their distinction between apparently-Lamarckian-but-actually Darwinian evolution, applies. I’d broaden this argument even further. I’d in fact suggest here that it might apply to the entire range of evolvability mechanisms that span the almost-continuous range between Darwinian and Lamarckian evolution (as surveyed by the authors in their previous essay on the subject [11]), and indirectly by us, too, more recently [56]. Inspecting each evolvability mechanism along the adaptation spectrum could reveal random, or biased, modes of Inheritance of Acquired adaptive Characters. Starting from stress induced mutagenesis (perhaps the most rudimentary deviation from purely Darwinian evolution), this phenomenon and process may or may not be biased towards certain genes (e.g., transcription coupled mutagenesis may favor mutations in genes depending on their current level of transcription). Only the latter flavor of stress induced mutagenesis might qualify, according to the current thesis, as truly Lamarckian. Going further into epigenetics (an important source of quasi-Lamarckian mode of evolution), at the protein level, prions are suggested, e.g., by Lindquist et al. to convey Lamarckian evolution [57], such as in the case of the ribosome release factor, Sup35, that when in prion form, allows the ribosome to read through STOP codons and exposed and translate otherwise latent genetic variability in genes’ 3’UTR. Consider a scenario when reading through a STOP codon were totally random, or when it was biased for certain genes, as suggested by several ribosome profiling studies. And still within epigenetics, consider DNA-template-free RNA replication, another potential mode of Lamarckian inheritance [58], perhaps there, too, RNA-dependent RNA polymerase could be selective to various degrees towards the RNAs that it selects to replicate. In HGT too, while most cases of foreign DNA uptake are probably random (and hence should be considered less Lamarckian than previously appreciated), bias is still certainly possible, either in cases the insert the DNA through homologous recombination, or in some cases due to apparent sensing of the DNA that takes place during the uptake itself [59]. Reverse transcription might also serve as a Lamarckian agent [56]. In this process, some of the genome’s genes may take a ride on an RT activity in the cell, and perhaps in some similarity to the I-E CRISPR-cas system discussed here, highly expressed genes are obviously more highly represented among the reverse transcribed genes in the genome [60], and on top of that, a possibility exists that certain RNAs are preferentially reversed transcribed beyond the level expected due to their expression level [61]. Thus, as recognized by Koonin and Wolf here, their distinction suggested here between truly Lamarckian processes, that selectively propagate an acquired trait, and an actually Darwinian process, that requires further selection to determine the fate of traits, is indeed broad and hence useful.

Authors’ response: *We appreciate these helpful and insightful comments of Dr. Pilpel and definitely agree that the variety of evolutionary phenomena, both genetic and epigenetic, that fit in different parts of the continuous range between ‘fully Lamarckian’ and ‘fully Darwinian’ is extremely broad. Various aspects of these phenomena, in particular the possible routes from epigenetics to genetics, have been discussed by one of us previously* [10, 62].

### Reviewer 2: Martijn Huynen, Radbaud University

I think the article provides a succint discussion of the Lamarckian aspects of the CRISPR system. By discussing the differences between systems I and II the authors zoom in to the subtleties that separate pure Darwinian and pure Lamarckian processes, and the continuum of mechanisms in between them.

Figure 2 actually does not show selection (which is an essential aspect of the Darwinian process) and it is not very apparent to me what the message is that Fig. 3 conveys. The text is clearer. That “Darwinian and Lamarckian modes of evolution are not fundamentally distinct” (abstract) remains a stretch, even if one can draw a continuum between them.

Authors’ response: *Fig.**2**was amended to explicitly indicate the selective steps. The purpose of Fig.**3**is to emphasize that Lamarckian mechanisms appear at a relatively advanced stage of the evolution of life as opposed to Darwinian and Wrightian modes of evolution that are intrinsic to replication processes. We agree that the quoted sentence (actually, not in the abstract but in the Conclusions) could be misleading and was unnecessary. We removed it in the revision and stick to the statement on the continuum of evolutionary mechanisms.*

### Reviewer 3: Bojan Zagrovic, University of Vienna

Using the CRISPR-Cas system, Koonin and Wolf present a series of arguments in support of the thesis that the strictly Darwinian and the strictly Lamarckian modes of evolution are actually just the extremes of a continuum of evolutionary modalities which exist in nature. The key question then is not which of the modalities is true and right, but rather, for a given system, what are the mechanisms that define the level of specificity of mutational processes that define it. The CRISPR-Cas system, for example, offers examples of both extremes. I find the arguments presented in the manuscript clear and compelling and the main conclusions well supported by the evidence, and in some way, almost expected. Namely, there exist a superficial (but, perhaps also in some ways enlightening) parallel with the current thinking on receptor-ligand binding in the structural biology community (see, for example, [63]). The long-accepted induced fit mechanism, in which the ligand causes the conformational change in the receptor leading to optimal binding, has been challenged by the conformational selection mechanism, in which the ligand only selects the binding-optimal conformer of the receptor from an ensemble of interconverting, pre-existing conformers. It has, however, recently become clear that many systems actually sit somewhere in between and that nature employs a full continuum of mechanisms defined by these two extremes. It is amusing to note that in this case, however, the more Lamarckian-type view (induced fit) actually represents the classical and the more widely accepted stance, while the Darwinian-type conformational selection took longer to be recognized. Finally, the fact that even for a phenomenon as elementary as ligand-receptor binding one sees mechanisms of a similar epistemological complexity as those concerning the much more complex evolutionary modes suggests a speculation that, contrary to what Koonin and Wolf propose, the elements of Lamarckian evolution might be found even in very simple, primordial systems with only rudimentary, primitive “repair” mechanisms.

Authors’ response: *We greatly appreciate these interesting comments of Dr. Zagrovic. The parallel between Lamarckian and Darwinian processes in evolution and modes of receptor-ligand binding seems enlightening but it is indeed a parallel or a simile because such binding is not a bona fide evolutionary process.*

1. It may be good to define protospacer (l. 93) – it is clear from the context, but for the readers who are not familiar with the CRISPR-Cas system, it might be helpful to give it an explicit definition. 2. I very much like the graphical illustrations of the concepts, but do think that a bit of simplification of the pictures might make them even more clear to read and interpret. For example, instead of showing 5 colored bars to depict genomes, perhaps 4 or even 3 might suffice. Also, the choice of colors could be such that acquisition of spacers (red) is visually in a stronger contrast with the rest.

Authors’ response: *An explicit definition of protospacers is given in the revision. In Figs.**1**and**2**, the 5 vertical bars depict the diversity of spacers in CRISPR arrays. Given that this diversity is high and the number of spacers in many cases large, we do not think that decreasing the number of bars would clarify the figures.*

## Abbreviations

Cas: CRISPR-associated (genes)
CRISPR: Clustered Regularly Interspered Palindromic Repeats
IAC: Inheritance of Acquired (adaptive) Characters
PAM: Protospacer Adjacent Motif
